# Silicon Valley new focus on brain computer interface: hype or hope for new applications?

**DOI:** 10.12688/f1000research.15726.1

**Published:** 2018-08-21

**Authors:** Stefan Mitrasinovic, Alexander P.Y. Brown, Andreas T. Schaefer, Steven D. Chang, Geoff Appelboom

**Affiliations:** 1 University College London Medical School, London, UK; 2 The Francis Crick Institute, London, UK; 3 Department of Neuroscience, Physiology and Pharmacology, University College London, London, UK; 4 Department of Neurosurgery, Stanford University Medical Center, Brighton, USA; 5 Byers Center for Biodesign, Stanford University School of Medicine, Brighton, USA

**Keywords:** Brain computer interface, brain machine interface, neuralace

## Abstract

In the last year there has been increasing interest and investment into developing devices to interact with the central nervous system, in particular developing a robust brain-computer interface (BCI). In this article, we review the most recent research advances and the current host of engineering and neurological challenges that must be overcome for clinical application. In particular, space limitations, isolation of targeted structures, replacement of probes following failure, delivery of nanomaterials and processing and understanding recorded data. ****Neural engineering has developed greatly over the past half-century, which has allowed for the development of better neural recording techniques and clinical translation of neural interfaces. Implementation of general purpose BCIs face a number of constraints arising from engineering, computational, ethical and neuroscientific factors that still have to be addressed. Electronics have become orders of magnitude smaller and computationally faster than neurons, however there is much work to be done in decoding the neural circuits. New interest and funding from the non-medical community may be a welcome catalyst for focused research and development; playing an important role in future advancements in the neuroscience community.

## Abbreviations and Acronyms

3D, Three-dimensional

BCI, Brain-Computer Interface

BMI, Brain-Machine Interface

CNS, Central Nervous System

CPU, Central Processing Unit

DPU, Decoding Processing Unit

ECoG, Electrocorticography

EEG, Electroencephalogram

fMRI, Functional Magnetic Resonance Imaging

PET, Positron Emission Tomography

PNS, Peripheral Nervous System

TPU, Tensor Processing Unit

## Introduction

In the last year, there has been an explosion of interest by entrepreneurs looking to become actively involved in developing devices to interact with the central nervous system. These have included the likes of Elon Musk (Neuralink Inc. California USA), Mark Zuckerberg (Facebook Inc. California, USA), Bryan Johnson (Kernel. California, USA) as well as dedicated startups such as Paradromics (San Jose, California, USA) or Cortera (Berkeley, California, USA), and even DARPA (Defense Advanced Research Projects Agency. Virginia, USA), spurred on in part by the BRAIN initiative ^1^. Each of these individuals and their respective companies share a particular focus in developing a robust brain-computer interface (BCI). We define BCI, for the purposes of this discussion, as a technological system designed to provide a stable mapping and modulation of activity within neural networks of the central nervous system. Therefore, at the very minimum, a working BCI will require both a physical interface to the brain (brain-machine interface; BMI) and computer systems that can process high bandwidth signals in real-time.

It is important to distinguish that there are very different engineering and neurological challenges between building BCIs for the peripheral nervous system (PNS) and central nervous system (CNS). In particular, space limitations for processing units, isolation of targeted structures, replacement of probes following failure, and delivery of nanomaterials *in vivo* ^2, 3^; for the purpose of this commentary we will focus on the CNS as this is an area of particular interest by the entrepreneurs highlighted above.

Understanding the information transfer and processing of the nervous system is one of the most urgent challenges faced by the biomedical community, with a plethora of academic and clinical applications, including better understanding of aging, neurodegenerative diseases and interfaces for prosthetics and implants. For example, recent advances in chronic neural recording devices have facilitated the willful control of robotic prosthetic limbs for the treatment of paralysis ^4^ and improved seizure prevention with chronic telemetry in refractory epilepsy ^5, 6^. There are many different kinds of potential BCIs that will each serve independent functions, however all systems must tackle three fundamental problems: how to accurately record information from relevant neural systems, how to decode such information, and how to stimulate and manipulate neuronal dynamics in an appropriate and meaningful way.

## Neural engineering progress

The origins of neural engineering stretch back to early attempts to record activity chronically in the 1950s when electrodes were implanted into the cortex of rhesus monkeys to measure electrical activity in the central nervous system ^7, 8^. Great innovations have been made in neural recording techniques, which have allowed the number of simultaneously recorded neurons to double approximately every 7 years ^9^, mimicking Moore’s law albeit at a much reduced rate ^10^. Early clinical applications of BMIs centered on the restoration of perceptions to patients with sensory deficits. One of the pioneering studies was the work on potential cochlear implants in the 1970s that eventually reached life-changing reality in the 1980s for patients ^11– 13^.

 In parallel to the development of the cochlear implant, researchers worked with the CNS by applying electrical current to the visual cortex of blind patients through grids of surface electrodes implanted over the visual cortex, thus developing visual prostheses ^14, 15^. These systems allowed blind subjects to learn to recognize simple visual objects ^16^. Neural engineering continued to improve with multi-channel neuronal recordings allowing owl monkeys ^17^ and later humans ^4^ to control two- and three-dimensional movements of a robot arm with multiple degree of freedom. Neuro-prosthetic research has undoubtedly benefited from these advances, but additional design parameters need to be included for effective long-term operation and clinical translation of neural interfaces.

While research in neural engineering has been steadily improving the bandwidth of BCI interfaces, the pace of this exponential increase falls far short of that seen in the silicon chip industry ^9^. At current pace, the goal set by DARPA of recording from 10 ^6^ neurons simultaneously would not be expected to be reached for around 80–100 years. Increasing interest and funding from members of Silicon Valley may prove to be a useful catalyst for the field and promote investigation of new applications of BCIs. For example, Facebook Inc. is investigating methods of non-verbal communication that will not require the virtual keyboards that are currently being used by patients with BrainGate ^18^.

## Challenges

Despite advances in recent years, implementation of general purpose BCIs faces a number of constraints arising from engineering, computational, ethical, and neuroscientific factors. The future success of BCI is often imagined as a function of the capability to produce multi-electrode arrays with a greater and greater density of recording sites. Here, we outline several other challenges that must be overcome in parallel if BCI is to become of more than limited interest. 

Perhaps the most immediate barrier to wider usage of BCI systems is the difficulty in implanting them. Non-invasive modalities, such as electroencephalogram (EEG) but also positron emission tomography (PET) and functional magnetic resonance imaging (fMRI) lack the spatial resolution to record detailed activity at the level of the neuronal circuit, and so can only be used for very simple low bandwidth (typically binary choice) interfaces. There is no technology currently available that can record an action potential without the need for major surgery, although research into less invasive endovascular electrodes ^19^ and surface electrocorticogram (ECoG) devices is ongoing ^20^. Furthermore, the quality of recordings obtained via implantable electrodes degrades over time due to a combination of gliosis ^21, 22^, neuronal depletion ^23, 24^, and degradation of the system itself ^22, 25, 26^. This tends to limit recording times to a period of months or a few years at most, although the use of compliant materials ^27^ or soft ultra-thin wires ^28, 29^ designed to reduce mechanical shear has shown promise in reducing these effects.

By definition, detecting neuronal signals constitutes only one half of the BCI. These signals must then be able to be communicated to a computer via either a wired or wireless connection. This poses further challenges, necessitating a tunneled wire through the cranium. Wireless systems avoid this challenge, but create a host of new problems in turn including available bandwidth, safety, and the need for an implantable battery – which may last only a few months powering a large BCI system ^30, 31^. To give a sense of the challenge here, we calculate that a 100,000-electrode system would require a communication protocol at least as fast as a Thunderbolt ^TM^ 3 connection (Apple, Inc. & Intel, Inc.), currently the fastest available consumer-level wired standard. The required bandwidth could be reduced drastically by on-chip processing, reducing the dimensionality of the data, but this in turn requires vastly more complex devices, limiting the number of electrodes per device, and greatly increasing its volume – a critical flaw in any proposed intracranial device. Furthermore, onboard processing of any kind poses serious and mostly unexplored challenges in terms of the energy dissipation required to maintain the device at body temperature so as not to cause thermal damage to the brain.

Current multi-electrode array systems offer up to around one thousand recording channels ^32^, in turn providing monitoring for hundreds of neurons from a single area ^33^, sufficient for the control of several univariate parameters. More general purpose BCI will require the sampling of tens if not hundreds of thousands of units, potentially from multiple cortical regions. This poses engineering and surgical challenges far beyond what is currently achievable.

Computational and data analysis challenges arise from the highly parallel nature of multiunit recordings. In general, there are four steps utilized to decode neural activity. Firstly, the signal must be filtered to remove extraneous noise. Secondly, spikes must be detected. Thirdly, these spikes must be ‘sorted’, typically by waveform, in order to be assigned to ‘units’ – putative single neurons. Lastly, the inferred population spike train must be decoded in order to provide a control signal. Whilst the first and second of these steps are essentially solved, for sufficiently high signal-to-noise systems, spike sorting is still an area of active research ^34, 35^, with no clear optimal solution, and often relies on semi-automated systems that require a great deal of human input to fine tune. Spike sorting may not be strictly necessary for the training of accurate decoders, as the raw spatiotemporal pattern of activity may suffice, but this may in turn reduce the dimensionality of the data.

Real-time processing of highly parallel recording systems remains a key challenge in the field. Promising technologies include a move away from general-purpose central processing units (CPUs) to application specific integrated circuits designed to perform a limited number of operations, such as Google’s tensor processing unit (TPU) or the graphical processing chips found in most computers. It is not unreasonable to suspect that the solution to decoding neural activity may lie in dedicated ‘decoding processing units’ (DPUs). 

The physical scalability of BCI systems also poses a profound challenge. The brain is a three-dimensional (3D) structure. Unlike silicon wafers, manufacturing devices with a complex 3D structure and including integrated electronics poses a particular problem. Furthermore, current designs of multi-electrode arrays are typically not well suited to rapid scalability, requiring extensive redesign for each generation of device.

Even if this problem can be overcome, it may seem intuitive that more units result in greater bandwidth, however, the distributed nature of cortical processing has actually shown to result in a decreasing marginal value of each additional unit in terms of information retrieval ^36^. Therefore, the common mantra that more units results in more information does not follow, at least not proportionally. We simply do not understand well enough the nature of distributed information representation and processing in the neocortex to be able to make more than a rudimentary estimate of what a particular sequence of activity might ‘mean’.

## Conclusion

The literature has shown large decades of neuroscience research efforts in developing tools to probe the signaling complexity of the nervous system, with several clinical applications being developed. Although orders of magnitude smaller and computationally faster than neurons, our electronics cannot mimic the complexity of neural systems. Current understanding of the function of neural circuits could be compared to trying to understand the internet by means of a few dozen well-placed potentiometers in the data centers of service providers. This is not to disparage the efforts of neuroscientists, far from it, but rather to underscore that decoding neural circuits ranks among the deepest and most complex contemporary endeavors, and it will not be solved overnight by Silicon Valley enthusiasm and zeal alone. However, we consider that many of the engineering challenges outlines above are amenable to focused research and development, particularly those surrounding miniaturization and parallelization of recording systems. We support the interest of entrepreneurs in placing their focus on the neuroscience community, and we look forward to the future advancements that will undoubtedly be realized in the coming years.

## Data availability

No data are associated with this article

## References

[1] The White House: Fact Sheet: BRAIN Initiative. ObamawhitehouseArchivesGov.2013 Reference Source

[2] Chen Y Liu L: Modern methods for delivery of drugs across the blood-brain barrier. *Adv Drug Deliv Rev.* 2012;64(7):640–65. 10.1016/j.addr.2011.11.010 22154620

[3] Chen R Canales A Anikeeva P: Neural recording and modulation technologies.Nature Publishing Group.2017;2: 16093. 10.1038/natrevmats.2016.93 PMC6707077 31448131

[4] Hochberg LR Bacher D Jarosiewicz B: Reach and grasp by people with tetraplegia using a neurally controlled robotic arm. *Nature.* 2012;485(7398):372–5. 10.1038/nature11076 22596161 PMC3640850

[5] Cook MJ O'Brien TJ Berkovic SF: Prediction of seizure likelihood with a long-term, implanted seizure advisory system in patients with drug-resistant epilepsy: a first-in-man study. *Lancet Neurol.* 2013;12(6):563–71. 10.1016/S1474-4422(13)70075-9 23642342

[6] Morrell MJ, RNS System in Epilepsy Study Group: Responsive cortical stimulation for the treatment of medically intractable partial epilepsy. *Neurology.* 2011;77(13):1295–304. 10.1212/WNL.0b013e3182302056 21917777

[7] Lilly JC: Electrode and cannulae implantation in the brain by a simple percutaneous method. *Science.* 1958;127(3307):1181–2. 10.1126/science.127.3307.1181 13555865

[8] Strumwasser F: Long-term recording' from single neurons in brain of unrestrained mammals. *Science.* 1958;127(3296):469–70. 10.1126/science.127.3296.469 13529005

[9] Stevenson IH Kording KP: How advances in neural recording affect data analysis. *Nat Neurosci.* 2011;14(2):139–42. 10.1038/nn.2731 21270781 PMC3410539

[10] Moore GE: Cramming more components onto integrated circuits. *Electronics.* 1965;114–7. Reference Source

[11] Wilson BS Dorman MF: Cochlear implants: a remarkable past and a brilliant future. *Hear Res.* 2008;242(1–2):3–21. 10.1016/j.heares.2008.06.005 18616994 PMC3707130

[12] Eddington DK: Speech recognition in deaf subjects with multichannel intracochlear electrodes. *Ann N Y Acad Sci.* 1983;405(1):241–58. 10.1111/j.1749-6632.1983.tb31637.x 6575648

[13] House WF: Cochlear implants. *Ann Otol Rhinol Laryngol.* 1976;85 suppl 27(3Pt2):1–93. 779582

[14] Dobelle WH Mladejovsky MG: Phosphenes produced by electrical stimulation of human occipital cortex, and their application to the development of a prosthesis for the blind. *J Physiol.* 1974;243(2):553–76. 10.1113/jphysiol.1974.sp010766 4449074 PMC1330721

[15] Brindley GS: Sensations produced by electrical stimulation of the occipital poles of the cerebral hemispheres, and their use in constructing visual prostheses. *Ann R Coll Surg Engl.* 1970;47(2):106–8. 5452658 PMC2387784

[16] Dobelle WH Quest DO Antunes JL: Artificial vision for the blind by electrical stimulation of the visual cortex. *Neurosurgery.* 1979;5(4):521–7. 10.1227/00006123-197910000-00022 534058

[17] Wessberg J Stambaugh CR Kralik JD: Real-time prediction of hand trajectory by ensembles of cortical neurons in primates. *Nature.* 2000;408(6810):361–5. 10.1038/35042582 11099043

[18] Pandarinath C Nuyujukian P Blabe CH: High performance communication by people with paralysis using an intracortical brain-computer interface. *eLife.* 2017;6: pii: e18554. 10.7554/eLife.18554 28220753 PMC5319839

[19] Oxley TJ Opie NL John SE: Minimally invasive endovascular stent-electrode array for high-fidelity, chronic recordings of cortical neural activity. *Nat Biotechnol.* 2016;34(3):320–7. 10.1038/nbt.3428 26854476

[20] Khodagholy D Gelinas JN Zhao Z: Organic electronics for high-resolution electrocorticography of the human brain. *Sci Adv.* 2016;2(11): e1601027. 10.1126/sciadv.1601027 28861464 PMC5569954

[21] Kozai TD Jaquins-Gerstl AS Vazquez AL: Brain tissue responses to neural implants impact signal sensitivity and intervention strategies. *ACS Chem Neurosci.* 2015;6(1):48–67. 10.1021/cn500256e 25546652 PMC4304489

[22] Polikov VS Tresco PA Reichert WM: Response of brain tissue to chronically implanted neural electrodes. *J Neurosci Methods.* 2005;148(1):1–18. 10.1016/j.jneumeth.2005.08.015 16198003

[23] Szarowski DH Andersen MD Retterer S: Brain responses to micro-machined silicon devices. *Brain Res.* 2003;983(1–2):23–35. 10.1016/S0006-8993(03)03023-3 12914963

[24] Kotzar G Freas M Abel P: Evaluation of MEMS materials of construction for implantable medical devices. *Biomaterials.* 2002;23(13):2737–50. 10.1016/S0142-9612(02)00007-8 12059024

[25] Ward MP Rajdev P Ellison C: Toward a comparison of microelectrodes for acute and chronic recordings. *Brain Res.* 2009;1282:183–200. 10.1016/j.brainres.2009.05.052 19486899

[26] Barrese JC Rao N Paroo K: Failure mode analysis of silicon-based intracortical microelectrode arrays in non-human primates. *J Neural Eng.* 2013;10(6):066014. 10.1088/1741-2560/10/6/066014 24216311 PMC4868924

[27] Scholten K Meng E: Materials for microfabricated implantable devices: a review. *Lab Chip.* 2015;15(22):4256–72. 10.1039/c5lc00809c 26400550

[28] Du ZJ Kolarcik CL Kozai TDY: Ultrasoft microwire neural electrodes improve chronic tissue integration. *Acta Biomater.* 2017;53:46–58. 10.1016/j.actbio.2017.02.010 28185910 PMC5512864

[29] Guitchounts G Markowitz JE Liberti WA: A carbon-fiber electrode array for long-term neural recording. *J Neural Eng.* 2013;10(4):046016. 10.1088/1741-2560/10/4/046016 23860226 PMC3875136

[30] Rajangam S Tseng PH Yin A: Wireless Cortical Brain-Machine Interface for Whole-Body Navigation in Primates. *Sci Rep.* 2016;6: 22170. 10.1038/srep22170 26938468 PMC4776675

[31] Kim S Bhandari R Klein M: Integrated wireless neural interface based on the Utah electrode array. *Biomed Microdevices.* 2009;11(2):453–66. 10.1007/s10544-008-9251-y 19067174

[32] Steinmetz NA Pachitariu M Burgess CP: Recording large, distributed neuronal populations with next-generation electrode arrays in behaving mice. *Neuroscience.* 2016.

[33] Obien ME Deligkaris K Bullmann T: Revealing neuronal function through microelectrode array recordings. *Front Neurosci.* 2015;8:423. 10.3389/fnins.2014.00423 25610364 PMC4285113

[34] Rossant C Kadir SN Goodman DFM: Spike sorting for large, dense electrode arrays. *Nat Neurosci.* 2016;19(4):634–41. 10.1038/nn.4268 26974951 PMC4817237

[35] Rey HG Pedreira C Quian Quiroga R: Past, present and future of spike sorting techniques. *Brain Res Bull.* 2015;119(Pt B):106–17. 10.1016/j.brainresbull.2015.04.007 25931392 PMC4674014

[36] Carmena JM Lebedev MA Crist RE: Learning to control a brain-machine interface for reaching and grasping by primates. *PLoS Biol.* 2003;1(2):E42. 10.1371/journal.pbio.0000042 14624244 PMC261882

