# Supplementary material for: Silicon Valley new focus on brain computer interface: hype or hope for new applications?
Source: F1000Res. 2018 Aug 21;7:1327. [Version 1] doi: 10.12688/f1000research.15726.1 (PMC6343225; doi:10.12688/f1000research.15726.1)
Supplement: Peer review history

# Peer review: Referee response for version 1

**DOI:** 10.5256/f1000research.17164.r40098

**Published:** 2019-01-21

**Authors:** Theresa M. Vaughan

**Affiliations:** 1 Department of Health, Wadsworth Center, New York State Department of Health, Albany, NY, USA

This article is a very good summary of the state of play in BCI research as of 2019. It will be of interest to the BCI and general neuroscience communities. 

1. Numerous funding sources with deep pockets, coming from very well established, and successful companies, start-ups, as well as the government signal that the time for BCI breakthroughs is clearly anticipated. 
2. It may be appropriate to mention some of the already met needs of some users, such as the various P300 Speller systems in use by patients with motor neuron diseases. This population lacks any other communication means, and recent studies with ALS subjects’ use of BCI in their homes have shown reasonable success.
3. The authors have touched on the major and significant challenges. The challenge of safe, easily deployed high density electrodes, disposed in some 3D configuration inside the brain is extremely great. The reliable readout of these electrodes, and their connection to some external computing means is similarly difficult. Integrating the computational means with the sensors is clearly desirable but very difficult.
4. For fifty years this reviewer has seen technologies compared to that of the transistor, which grew from transistor radios with seven individual transistors, to 100 million transistors per square millimeter today. Approximately a 5e6 fold improvement. 
5. Unfortunately no other technology has had a similar arc, and BCI is unlikely to be similarly blessed with inexpensive scalable improvements.
6. Some comment on the possibility of using Artificial Intelligence of the type currently in use to learn, master and dominate games of chess and GO.
7. Recommend indexing.  

I have read this submission. I believe that I have an appropriate level of expertise to confirm that it is of an acceptable scientific standard.

# Peer review: Referee response for version 1

**DOI:** 10.5256/f1000research.17164.r40102

**Published:** 2018-11-15

**Authors:** Ujwal Chaudhary

**Affiliations:** 1 Wyss-Center for Bio- and Neuro-Engineering, Geneva, Switzerland; 2 Institute of Medical Psychology and Behavioural Neurobiology, University of Tübingen, Tübingen, Germany

The intention and the action performed to support the intention of the human-being is supported by the impeccable coordination of the peripheral nervous system (PNS) and the central nervous system (CNS). Any disruption in this coordination, for example dysfunction of afferent or efferent pathways or injury in the spinal cord or neurological disorders affecting the functioning of brain, affects the normal functioning of the human body. The individual becomes paralyzed and is unable to perform the simple day to day activities of walking or talking. Brain computer interfaces (BCIs) have been developed to help such individuals, where BCIs aim to bypass the dysfunctional pathways and interface the external mechanical or electrical devices with the functioning brain of an individual. Both non-invasive and invasive BCIs have been developed: in non-invasive BCIs non-invasive neuroimaging techniques are being used to acquire brain signals from the surface of the scalp while in invasive BCIs electrodes are placed on the cortical surface of the brain or inserted in the brain. The invasive technique where the electrodes known as microelectrodes are inserted in the brain records spike signals either from a single neuron, known as single unit activity (SUA) or from a group of neurons, known as local field potentials (LFPs). 

 In this article the authors have done a great job in summarizing the technical challenges faced by researchers during recording neural signals invasively and have discussed the different approaches developed to solve these problems. Given the recent media interest in the application of BCI as a tool for the means of communication and rehabilitation of paralysed people, several entrepreneurs have invested huge resources in developing BCI. The authors have applauded such efforts and have presented a bright outlook on the effect of such interests on the development of BCI for real world applications.

I have read this submission. I believe that I have an appropriate level of expertise to confirm that it is of an acceptable scientific standard.

# Peer review: Referee response for version 1

**DOI:** 10.5256/f1000research.17164.r40099

**Published:** 2018-11-06

**Authors:** Jeffrey V. Rosenfeld, Yan Tat Wong

**Affiliations:** 1 Monash Institute of Medical Engineering, Monash University, Melbourne, Vic, Australia; 2 Department of Surgery, Monash University, Clayton, Australia; 3 Department of Neurosurgery, Alfred Hospital, Melbourne, Australia; 4 Department of Physiology, Monash University, Clayton, Australia; 5 Department of Electrical and Computer Systems Engineering, Monash University, Clayton, Australia

This is a concise review by Mitrasinovic *et al.* concerning the evolution of Brain Computer Interfaces (BCIs), the current ‘state-of-the art’ of BCIs and the future challenges particularly in relation to electrode design, electrode placement and signal processing. 

 It is not immediately apparent to us why Silicon Valley is in the title. More should be made of this in the introduction. Specifically, in what areas do the authors believe that Silicon Valley can help advance BCIs to clinical reality? Are the private entrepreneurs mentioned in paragraph 1 based in Silicon Valley? A greater review of companies involved in BCI research may even be warranted. There are many research groups outside Silicon Valley where advances in computer and electronic engineering for BCIs are also taking place.

 Nanomaterials are mentioned in paragraph 1 but with no explanation as to how these are used in BCIs or why they would replace materials already in use. The discussion on nanomaterials could be moved to the section discussing improvements in electrode design.

 While recording electrode numbers have roughly doubled every seven years, is this the case for BCIs? The challenges in recording acutely from non-human primates with non-FDA approved electrodes is very different from the goal of BCIs in humans. At a minimum this difference should be highlighted to not give the readers an unrealistic view of progress.

 The difference between peripheral and central nervous system is highlighted but what about the major differences between non-invasive EEG platforms and invasive implanted electrodes on the surface or penetrating the brain? This should also be highlighted. There is rapid development of non-invasive EEG recording interfaces with the brain which avoid the inconvenience, risks and costs of surgical implantation. Will advances in signal processing increase the accuracy of these non-invasive BCIs and lessen the applications or need for implanted BCIs? It is difficult to imagine how EEG interfaces could compete with the implanted BCIs on the basis of the volume, precision and reliability of the information being transferred.

 PET and fMRI are mentioned as modes of BCI in addition to EEG.  These are not relevant to developing clinically- and commercially-relevant BCIs for ambulant individuals and we suggest that these modalities be deleted. At present the only way to activate neurons noninvasively is with transcranial magnetic stimulation (TMS). 

  

- Paragraph 2, page 4: The description of detecting neural signals only being “one half of a BCI” seems a little oversimplified. Not only do you need to record neural signals and decode them but next you need to use these signals to control an output such as a cursor on a screen or robotic limb and then also provide accurate feedback to the patient via electrical stimulation or other means. These challenges should be discussed.
- Paragraph 2, page 4: We would submit that wired implanted BCIs with a connector penetrating the scalp have no future as a permanently implanted device because of infection risk and inconvenience. Implanted BCIs must become wireless if they are to have any physician or patient uptake. Wireless devices are already described, for example in Lowery *et al.* (2015 ^1^), Rajangam *et al.* (2016 ^2^) and Vansteensel *et al.* (2016 ^3^). We agree there are challenges as the number of electrodes increase. 
- Paragraph 3, page 4: The number of electrodes required to adequately perform certain tasks is not known. Detailed vision, speech processing and fine motor control and the encoding and manipulation of memory would likely require significantly more electrodes than are currently available. However, vast increases in electrode numbers may not be required for all BCIs. The challenges of recording from ever increasing numbers of neurons has been laid out, but the challenges in basic neuroscience in understanding the basic coding of neurons in controlling movement should also be highlighted, for example, little is still known about the coding of control for grasping in the dorsal and ventral pre-motor cortices so this lack of knowledge affects our ability to extract information from these recorded populations of neurons.
- Paragraph 4, page 4: The four steps that you outline to decode neural signals are focused on spike decoding, whereas the paragraphs before outline techniques that will not result in spike recordings, e.g. EEG, ECOG, endovascular devices. A broader description on decoding algorithms that includes the use of low frequency continuous signals such as the Local Field Potentials (LFP) possibly separated in step called “feature extraction” is needed. Before neural signals can be decoded, algorithms also need to be trained which is not a trivial problem for the target patients.
- Paragraph 2, column 2, page 4: A large push in BCIs is getting the hardware necessary to be small enough and run on low power to allow patients to be mobile. In the description of parallel processing and Central Processing Units (CPUs), this challenge should be discussed.
- The future design of BCIs using light or magnetic energy as an alternative to electricity could also be included in the discussion.
- Mention could also be made of the surgical risk of implantation which includes haemorrhage, epilepsy and infection. The mitigation of risk needs to be factored in to the design of the devices and included in the informed consent process. It is important for physicians to work alongside engineers and scientists in the development of BCIs so that they are as safe and practical as possible.
- A mention of the many ethical challenges such as informed consent, agency, stigma, equity, neural enhancement, privacy and security of data is important in a general review such as this. For example, there are major ethical challenges to apply BCIs in severely disabled individuals to allow communication or control of assist devices such as locked-in syndrome or advanced amyotrophic lateral sclerosis (ALS).

We have read this submission. We believe that we have an appropriate level of expertise to confirm that it is of an acceptable scientific standard, however we have significant reservations, as outlined above.

## References

[1] LoweryAJRosenfeldJVLewisPMBrowneDMohanABruntonEYanEMallerJMannCRajanRRosaMPritchardJ: Restoration of vision using wireless cortical implants: The Monash Vision Group project. *Conf Proc IEEE Eng Med Biol Soc*.2015: 10.1109/EMBC.2015.7318543 1041-4 10.1109/EMBC.2015.7318543 26736443

[2] RajangamSTsengPHYinALehewGSchwarzDLebedevMANicolelisMA: Wireless Cortical Brain-Machine Interface for Whole-Body Navigation in Primates. *Sci Rep*.2016;6: 10.1038/srep22170 22170 10.1038/srep22170 26938468PMC4776675

[3] VansteenselMPelsEBleichnerMBrancoMDenisonTFreudenburgZGosselaarPLeindersSOttensTVan Den BoomMVan RijenPAarnoutseERamseyN: Fully Implanted Brain–Computer Interface in a Locked-In Patient with ALS. *New England Journal of Medicine*.2016;375(21) : 10.1056/NEJMoa1608085 2060-2066 10.1056/NEJMoa1608085 PMC532668227959736
